# Medial Prefrontal Cortex Theta Burst Stimulation Improves Treatment Outcomes in Alcohol Use Disorder: A Double-Blind, Sham-Controlled Neuroimaging Study

**DOI:** 10.1016/j.bpsgos.2022.03.002

**Published:** 2022-03-15

**Authors:** Daniel M. McCalley, Navneet Kaur, Julia P. Wolf, Ingrid E. Contreras, Sarah W. Book, Joshua P. Smith, Colleen A. Hanlon

**Affiliations:** a Department of Psychiatry and Behavioral Sciences, Medical University of South Carolina, Charleston, South Carolina; b Department of Neurosciences, Medical University of South Carolina, Charleston, South Carolina; c Department of Cancer Biology, Wake Forest School of Medicine, Winston-Salem, North Carolina

**Keywords:** Alcohol use disorder, Cue reactivity, fMRI, Medial prefrontal cortex, Theta burst stimulation, Transcranial magnetic stimulation

## Abstract

**Background:**

Alcohol use disorder (AUD) is associated with elevated brain response to cues. Recent studies have suggested that theta burst stimulation (TBS) to the medial prefrontal cortex (MPFC) can decrease reactivity to cues in a transdiagnostic manner. The goal of this clinical trial was to evaluate the effect of continuous TBS as a tool to decrease drinking behavior and brain reactivity to alcohol cues among individuals with AUD.

**Methods:**

A total of 50 individuals with AUD were recruited from an intensive outpatient treatment program. Using a randomized, double-blind, sham-controlled design, participants received 10 sessions of continuous TBS (left frontal pole, 1 session/10 days, 110% resting motor threshold, 3600 pulse/session, cue provocation before and during session). Brain reactivity to alcohol cues was acquired at four time points: at baseline and after all TBS sessions (1 month, 2 months, and 3 months).

**Results:**

Overall, 80% of the participants completed all TBS sessions. Individuals who received real TBS were 2.71 times more likely to remain enrolled in the study after 3 months and 3.09 times more likely to remain sober 3 months after treatment initiation. Real TBS also led to a significantly greater reduction in brain reactivity to alcohol cues, specifically a reduction in MPFC-striatum and MPFC-insula connectivity 2 and 3 months after TBS treatment.

**Conclusions:**

Ten days of MPFC TBS is well tolerated, reduces drinking, and decreases brain reactivity to alcohol cues for up to 3 months after treatment initiation. These results pave a critical next step in the path toward developing transcranial magnetic stimulation as an intervention for AUD and disorders associated with elevated cue reactivity.

Alcohol dependence is an intransigent health problem that affects over 1 billion individuals worldwide, levying a financial burden to society similar to that of cancer. Alcohol use disorder (AUD) affects a wide variety of individuals (adolescents to senior citizens, low to high socioeconomic status). All of these populations are united by a common feature—elevated behavioral and brain reactivity to environmental cues for alcohol, a common cause of relapse (1, 2, 3, 4, 5, 6). The brain regions most commonly engaged by alcohol cues include the medial prefrontal cortex (MPFC), ventral and dorsal striatum, anterior cingulate cortex (ACC), and anterior insula (1,5, 6, 7, 8, 9).

Recently, there is growing interest surrounding the use of noninvasive neuromodulation of these brain regions as a unique treatment tool for AUD (10,11). To date, there have been 17 studies to evaluate the efficacy of transcranial magnetic stimulation (TMS) as therapeutic option to decrease drinking. The majority of these studies have focused on increasing activity within the dorsolateral prefrontal cortex (DLPFC), a brain region involved in executive control. An alternative approach, however, is to decrease activity within regions associated with alcohol cue reactivity (e.g., MPFC, striatum, insula, ACC). For example, a single session of continuous theta burst stimulation (cTBS) applied to the left frontal pole (FP1) of the MPFC decreases brain reactivity to alcohol and drug cues within the frontal-striatal and frontal-insular circuits (12). Therefore, the next step in this treatment development pipeline is to determine if multiple sessions of cTBS to the FP1 decrease brain reactivity to alcohol cues as well as alcohol consumption.

In this article we report the results of a randomized, double-blind, sham-controlled trial evaluating cTBS to the left FP1 as a tool to decrease alcohol use and brain reactivity to alcohol cues for up to 3 months after treatment initiation. The primary aims of this study were to evaluate the 1) feasibility and 2) efficacy of FP1 TBS as a tool to improve retention and relapse rates among individuals engaged in an intensive outpatient treatment program, as well as 3) the effects of this intervention on brain reactivity to alcohol cues. The scientific rationale was based on a conceptual model that TBS to the FP1 decreases drug cue–induced activity in the MPFC, ACC, insula, and striatum, key nodes of the salient reward network (13). To determine if the TBS intervention was, in fact, modulating these circuits, functional neuroimaging data were obtained for all individuals at baseline, 1, 2, and 3 month time points.

## Methods and Materials

### Participants and Procedures

#### Participants

All experimental protocols were reviewed and approved by the Medical University of South Carolina Institutional Review Board and performed in accordance with the Declaration of Helsinki on Ethical Principles for Medical Research. Each participant provided written documentation of informed consent prior to enrolling in the study. A total of 50 treatment-seeking individuals with AUD (30 men, 20 women; 45.9 ± 11.7 years old) were recruited from the Intensive Outpatient Program at the Center for Drug and Alcohol Problems at Medical University of South Carolina in Charleston, South Carolina. In the Center for Drug and Alcohol Problems Intensive Outpatient Program, participants attend daily group therapy sessions with clinicians trained in several different modalities of evidence-based treatment for AUD (e.g., motivational enhancement therapy, cognitive behavioral therapy, twelve step facilitation, and acceptance and commitment therapy). In addition, alumni from this program are invited to attend monthly continuity visits. As part of this program, urine screens are performed intermittently to evaluate the presence of ethyl glucuronide (ETG), a biomarker for recent alcohol consumption. Exclusion criteria for this clinical trial included current or prior dependence on prescription or psychoactive drugs other than nicotine (*n* = 26), history of head injury with loss of consciousness, unstable medical illness, pregnancy or breastfeeding, ferromagnetic metal in the body, history of seizures, and a Clinical Institute Withdrawal Assessment score > 5. Of the 50 individuals, two were taking naltrexone. See the Supplement for more information on the Center for Drug and Alcohol Problems and exclusion criteria.

#### Experimental Timeline

**Figure 1:**
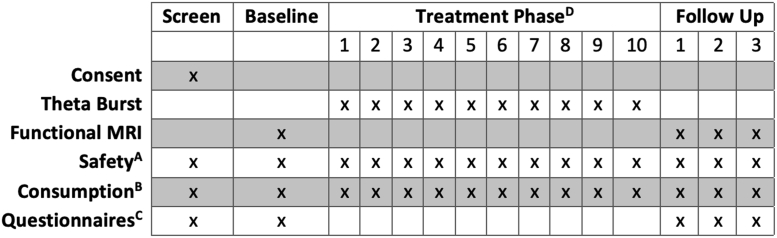
Experimental design. All participants were enrolled in the study during the first week of Intensive Outpatient Programming. During weeks 2 and 3 of this program, participants were randomized to receive 10 days of real or sham theta burst stimulation. ^A^At each visit, various safety measurements were performed, including urine screening for other drugs of abuse that might affect the motor threshold, changes in medical history, and Clinical Institute Withdrawal Assessment. Transcranial magnetic stimulation tolerability and any adverse events were also collected. ^B^Self-reported drinking was collected at each study visit for all days since the previous study visit, including the follow-up periods. Quantitative urine metabolites for ethyl glucuronide were taken intermittently during the treatment and follow-up phases. ^C^Alcohol Use Disorders Identification Test scores (baseline only), Beck Depression Inventory-II, State-Trait Anxiety Inventory, Obsessive-Compulsive Drinking Scale, and Alcohol Urge Questionnaire. ^D^Left frontal pole, 1 session/10 days, continuous theta burst stimulation, 3600 pulses/session, cue provocation before and during session, 110% resting motor threshold. MRI, magnetic resonance imaging.

Following consent and screening, eligible individuals were randomized to receive 10 sessions of real or sham TBS (Figure 1). Average time to completion of the 10 sessions was 14.80 ± 4.90 business days. Functional magnetic resonance imaging (MRI) scans were collected at baseline (before TMS) and 1, 2, and 3 months after treatment initiation. The following clinical assessments were collected: Timeline Followback for alcohol use (60 days at intake; daily thereafter; 30 days at the follow-ups) (14), Obsessive-Compulsive Drinking Scale (OCDS) (15), Alcohol Urge Questionnaire (AUQ) (16), Beck Depression Inventory-II (17), State-Trait Anxiety Inventory (18), and the Barratt Impulsiveness Scale (19). In addition, the Alcohol Use Disorders Identification Test (20) and Fagerström Test for Nicotine Dependence (21) were taken at baseline.

#### Continuous TBS

TBS procedures were performed using a figure-of-eight Cool B-65 A/P coil (Magventure). At baseline, individual resting motor threshold was determined using Parameter Estimation by Sequential Training, an automated algorithm used to determine TMS thresholds (22). The TMS coil was positioned at the FP1 using the standard electroencephalography 10–20 landmark location. TBS was administered at 110% of each participant’s resting motor threshold. Pulses were administered in a burst-firing pattern (3 pulse burst, 50 Hz; 5 Hz [200-ms] interburst intervals; 1800 pulses/train with a 60-second intertrain interval [3600 pulses/day]). To enhance tolerability of FP1 TBS, stimulation intensity was gradually escalated from 30% to 110% of resting motor threshold over the first 30 seconds of each train.

This double-blind study used the MagVenture MagPro integrated active-sham system, wherein a USB key coded with participant numbers was inserted into the machine prior to each participant’s visit and electrodes (Natus Inc.) are placed on the left frontalis muscle under the coil. During stimulation, current was passed through the surface electrodes at an intensity scaled to their motor threshold. Following each TMS treatment, individuals were asked to note whether they received real or sham and their level of confidence (Likert scale, 1–10).

#### Behavioral Priming Before and During TBS

Previous studies have demonstrated that exposure to cues prior to TMS can amplify treatment response (23, 24, 25, 26). As previously described (12), before delivery of TMS, individuals were instructed to recall the last time they had used alcohol. The staff member asked a standard set of questions tailored to the participant’s history, guiding them to describe the sensory aspects of the experience (environment, social setting, the way the beverage made them feel). The staff member instructed the participant to “keep thinking about alcohol and the negative aspects of how it makes you feel” during the session.

#### Structural and Functional Neuroimaging

High-resolution, T1-weighted structural scans (Inversion recovery, 3D spoiled gradient echo, 1.0 × 1.0 × 1.0, field of view: 256 mm, section thickness: 1.0 mm, no gap, in-plane resolution: 256) and T2∗-weighted images (multislice, gradient echo-planar sequence, repetition time = 2200 ms; echo time = 35.0 ms; 3.0 × 3.0 × 3.0 mm; field of view: 192 mm; resolution: 64) were collected throughout the study.

#### Alcohol Cue Functional MRI Task

As previously reported (12,27,28), our alcohol cue task was administered through E-Prime 2 software (Psychology Software Tools, Inc.). Images were presented within a block design (12 min: 24-s blocks, 4.8 s/image) wherein blocks represented four conditions presented in a pseudorandom order: alcohol, neutral beverages, blur (matching substance images in color and hue), and rest (fixation cross). The task was described to participants prior to the scan, and participants were asked to rate their level of alcohol craving following each block of images using an MRI-compatible hand pad (Likert scale, 1–5).

### Neuroimaging Analysis

#### Scalp-to-Cortex Distance

Scalp-to-cortex (STC) distance was extracted from FP1 for each subject using SIMNIBS v.3.2.1 (29). Individual STC measurements, representing the shortest distance between the cortex and the area immediately underlying the TMS coil, were extracted from SIMNIBS’ standard output when modeling electrical fields at the FP1.

#### Within-Subject Analysis

All functional MRI data were preprocessed using SPM12 (Wellcome Department of Cognitive Neurology) implemented in MATLAB 2017b (The MathWorks, Inc.). Standard preprocessing steps included segmentation, skull stripping, field map correction, motion correction, and normalization to MNI-152 space. See the Supplement for expanded details.

The analytic plan for this dataset was based upon a previous publication wherein we demonstrated that a single session of cTBS to the FP1 decreased functional connectivity to cues in the cingulate, striatum, and insula (12). The purpose of this study was to determine if multiple sessions of TMS also decreased brain reactivity to alcohol cues and if these decreases were durable for 2 to 3 months after TMS.

We used the Conn functional connectivity toolbox (version 20.b) to evaluate functional connectivity (30). A weighted general linear model and region of interest (ROI)-to-ROI analyses were performed on the data from baseline and 1-, 2-, and 3-month follow-up visits. Brain ROIs were restricted to those identified by Kearney-Ramos *et al.* (12). The seed ROI, FP1, was constructed using a 20-mm brain-masked region located at the FP1 target (10–20 system).

Other ROIs in the analysis included: the left and right insula (AAL atlas: 29_Insula_L, 30_Insula_R), bilateral anterior cingulate cortex (AAL atlas: 31_Cingulum_Ant_L, 32_Cingulum_Ant_R), left and right dorsal striatum (Oxford-GSK_Imanove connectivity atlas), left and right ventral striatum (Oxford-GSK_Imanove connectivity atlas), and the superior occipital cortex (AAL atlas: 49_Occipital_Sup_L; control region).

Fisher’s transformed correlation coefficients (*z* scores) were extracted between FP1 (stimulation site) and each ROI. Functional connectivity associated with alcohol cue blocks was compiled for each participant at all visits.

#### Between-Group Analysis

Data from above were entered into a group level mixed-effects general linear model (SPSS) to determine the effect of treatment, time, and ROI on change in FP1 functional connectivity relative to baseline. The model included covariates for individual STC distance, gender, state anxiety score (State-Trait Anxiety Inventory, trait subscale), depression score (Beck Depression Inventory-II), and AUD severity (Alcohol Use Disorders Identification Test). Estimated marginal means of the main effects and interactions were quantified.

Prior to group-level multivariate regression, statistical outliers were identified using the SPSS boxplot tool. Boxplots were constructed for change in functional connectivity for each ROI. Extreme outliers (datapoints > [third quartile + (3 × interquartile range)] and datapoints < [first quartile − (3 × interquartile range)]) were excluded from analysis, leaving 97.4% of all data included in analysis (Table S1).

#### Secondary Analysis

In the interest of facilitating future work in this area, wherein investigators may require effect sizes to power larger, more definitive clinical trials, we performed a post hoc analysis. We evaluated the influence of real versus sham TBS on functional connectivity at each timepoint for each ROI. Effect sizes (Hedges’ *g*, weighted for different sample sizes) were interpreted as follows: small-medium effect size, *g* < 0.5; medium-large effect size 0.5 < *g* < 0.8; large effect size *g* > 0.8 (31).

### Behavioral Analysis

#### Study Enrollment

Standard odds ratios (ORs) were calculated at each follow-up visit to assess the likelihood of attendance following real or sham MPFC TBS.

#### Sobriety

One of the primary goals of this experiment was to determine if 10 sessions of cTBS to the FP1 improved the likelihood of sobriety in treatment seeking individuals with AUD. We collected daily self-reported drinking from participants (Timeline Followback) as well as intermittent quantitative ETG measurements collected while they were enrolled in the intensive outpatient treatment program. All individuals in the per-protocol sample with at least 1 heavy drinking day in the 30 days before TMS V1 (32 of 50) were included in the analysis. For each time point, we compiled the number of drinking days and the number of heavy drinking days (women: ≥4 and men: ≥5 standard drinks/day) in the 30 days prior to that time point. The self-report measurements were cross-referenced with outcomes from urine ETG (wherein either a positive self-report or a positive ETG level [>100] was considered a drinking day). The odds of drinking (any drinking days in the previous 30 days) were calculated using standard ORs. To deal with missing data from a statistical perspective, the last observation carry forward method was used (32). This is a common statistical technique in longitudinal clinical trials likely most appropriate when missing data is not equally distributed in the treatment and sham group (e.g., missing not at random) (33).

#### Change in Behavioral Assessments of Interest

Mixed-effects general linear models (time × treatment) were computed for secondary outcomes including change in OCDS and AUQ. Covariates including baseline score, individual STC distance, and gender were included in the model. Effect sizes reflecting the difference between groups at each follow-up visit were calculated.

## Results

### Baseline Demographics and Behavior

There were no significant differences between groups at baseline, with the exception of education (Table 1).

**Table 1:** Participant Demographics

	Real, *n* = 26	Sham, *n* = 24
Demographics		
Age, years	45.7 ± 11.5	46.2 ± 12.1
Education, years	15.9 ± 1.7	14.7 ± 2.0
Gender, *n*	10 F, 16 M	10 F, 15 M
Ethnicity, *n*	0 H, 26 NH	1 H, 22 NH, 1 NA
Race, *n*	3 AA, 23 W	1 A, 4 AA, 19 W
TMS Parameters		
RMT, % MSO	47.8 ± 8.6	51.0 ± 8.1
Drinking and Craving		
AUDIT	25.9 ± 5.7	26.7 ± 5.3
Consumption	10.2 ± 1.5	10.0 ± 2.3
Dependence	6.8 ± 2.8	7.5 ± 2.3
ARPS	8.9 ± 3.7	9.1 ± 3.7
OCDS	15.6 ± 6.1	10.8 ± 6.1
Obsessive	6.6 ± 2.9	5.3 ± 3.3
Compulsive	9.1 ± 4.3	5.3 ± 3.5
Days Since Last Drink	11.0 ± 10.4	14.4 ± 9.5
Drinking Days (Last 30 Days)	10.9 ± 8.1	9.3 ± 8.5
Heavy Drinking Days (Last 30 Days)	8.9 ± 8.1	7.8 ± 8.0
AUQ	23.8 ± 14.7	15.4 ± 9.0
AUD-Associated Comorbidity		
BDI	16.9 ± 12.01	15.4 ± 10.8
STAI (State)	41.0 ± 14.9	43.3 ± 14.9
STAI (Trait)	45.5 ± 14.1	47.0 ± 14.7
BIS	67.7 ± 13.5	66.3 ± 13.6
Smoking		
Current smoking status, *n*	17 S, 9 NS	12 S, 13 NS
FTND	3.7 ± 2.9	3.7 ± 3.0

There was no difference in demographic variables between the real and sham group, with the exception of education. Values represent mean ± SD unless otherwise specified.

A, Asian; AA, African American; ARPS, Alcohol-Related Problems Scale; AUD, alcohol use disorder; AUDIT, Alcohol Use Disorders Identification Test; AUQ, Alcohol Urge Questionnaire; BDI, Beck Depression Inventory-II; BIS, Barratt Impulsiveness Scale; F, female; FTND, Fagerström Test for Nicotine Dependence; H, Hispanic; M, male; MSO, machine stimulator output; NA, no answer; NH, non-Hispanic; NS, nonsmoker; OCDS, Obsessive-Compulsive Drinking Scale; RMT, resting motor threshold; S, smoker; STAI, State-Trait Anxiety Inventory; TMS, transcranial magnetic stimulation; W, White.

### Sham Integrity

Of the 469 survey responses over the full study, the accuracy of correctly guessing was 50.11%, confirming the integrity of the sham. Participants endorsed moderate confidence in their decision (6.7 ± 2.5 on a 1–10 scale).

### Enrollment and Sobriety

**Figure 2:**
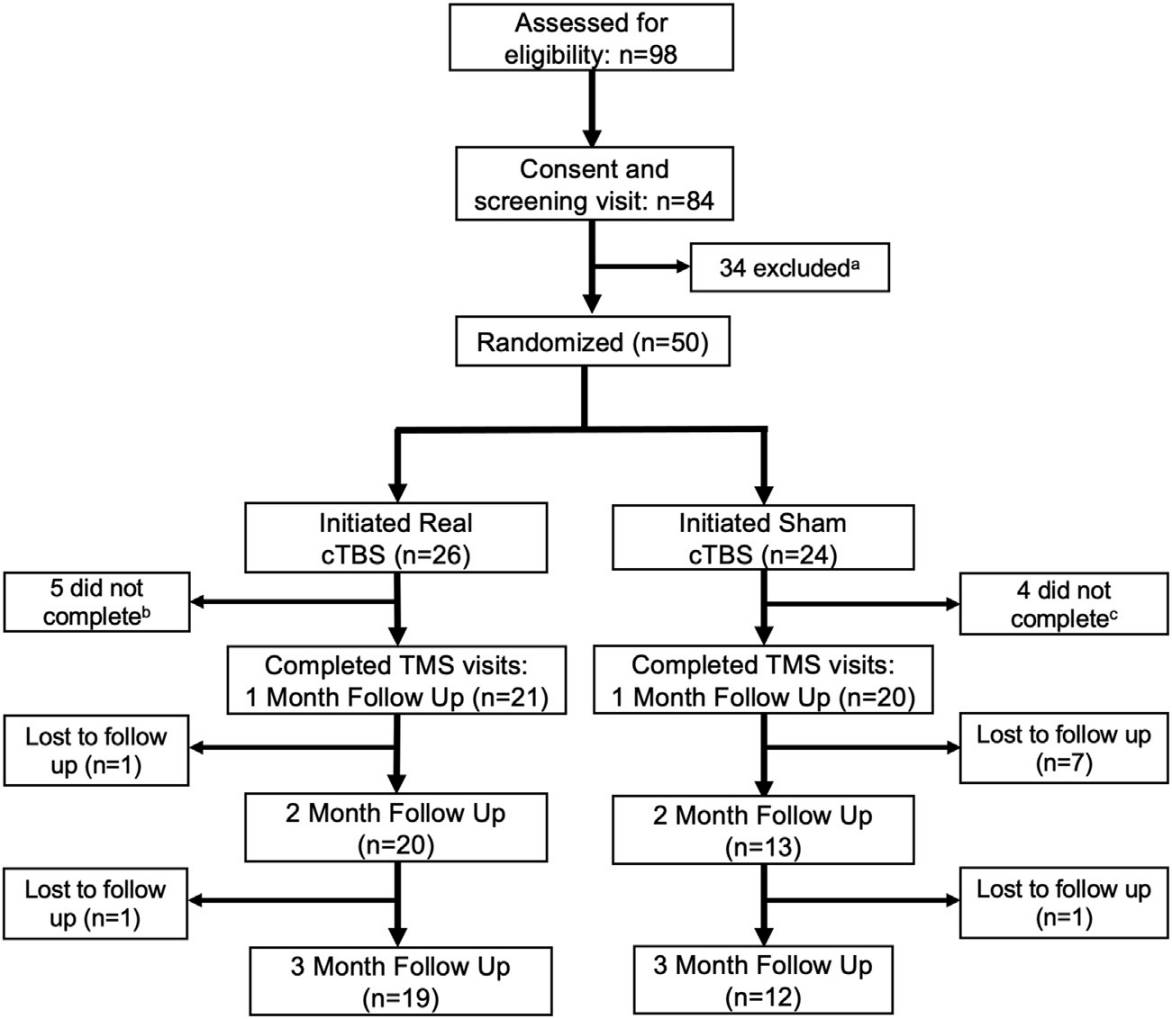
CONSORT diagram showing flow of participants through the theta burst stimulation (TBS) experiment. ^a^A total of 34 participants were excluded after consent and screening due to recent abuse of illicit substances other than marijuana (*n* = 23), no contact after screening visit (*n* = 6), met criteria for substance dependence on Xanax (*n* = 1), not eligible due to age (*n* = 1), participant withdrawn from outpatient program (*n* = 1), participant currently taking prescription opiates (*n* = 1), or failed metal safety screening (*n* = 1). ^b^Five subjects were lost to follow-up at varying stages of real TBS treatment (three sessions completed, *n* = 1; six sessions completed, *n* = 1; seven sessions completed, *n* = 2; nine sessions completed, *n* = 1). ^c^Four subjects were lost to follow-up at varying stages of sham TBS treatment (two sessions completed, *n* = 1; three sessions completed, *n* = 1; four sessions completed, *n* = 1; seven sessions completed, *n* = 1). cTBS, continuous TBS; TMS, transcranial magnetic stimulation.

**Figure 3:**
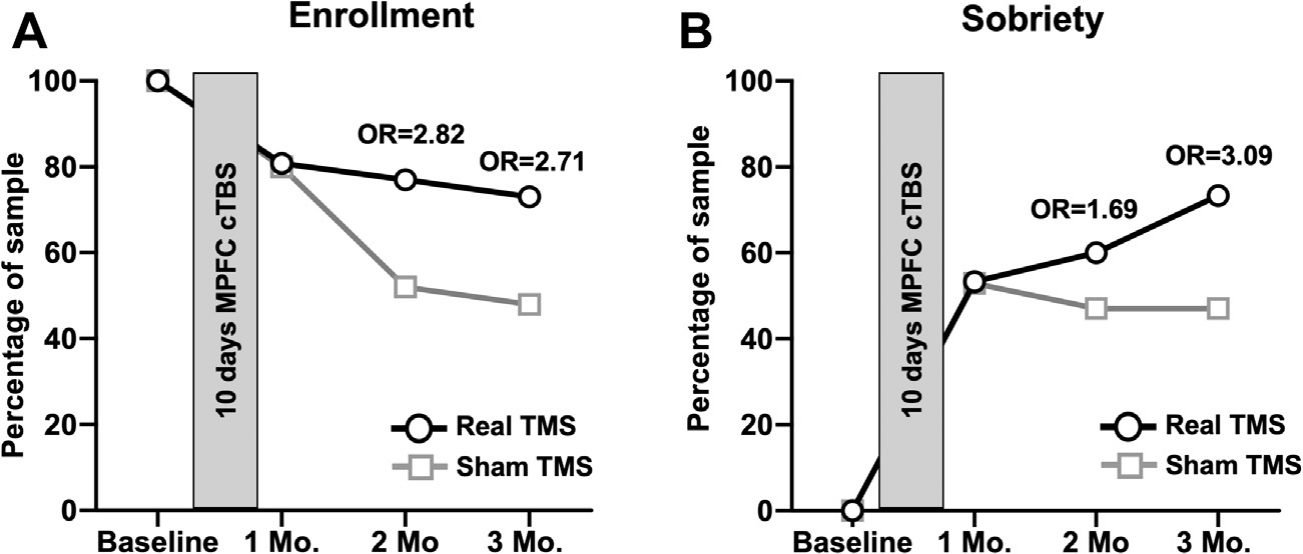
Study enrollment and sobriety following real vs. sham theta burst stimulation (TBS). Individuals in the real TBS group (black lines, circles) were more likely to remain enrolled in the study and more likely to remain sober than the sham group (gray lines, squares). **(A)** Percentage enrolled (1 mo: real, 80.1%; sham, 80.0%; 2 mo: real, 77.0%; sham, 52.0%; odds ratio [OR] = 2.82, *z* = 1.672, *p* = .1; 3 mo: real, 73.1%; sham, 48.0%; OR = 2.71, *z* = 1.66, *p* = .1). **(B)** Percentage of individuals remaining sober (1 mo: real, 31.0%; sham, 42.9%; 2 mo: real, 45.5%; sham, 42.9%; 3 mo: real, 72.7%; sham, 47.6%; OR = 2.93, *z* = 1.66, *p* = .1). cTBS, continuous TBS; MPFC, medial prefrontal cortex; TMS, transcranial magnetic stimulation.

The CONSORT (Consolidated Standards of Reporting Trials) diagram (Figure 2) describes the details regarding enrollment and retention in the study. Although there was not a statistically significant difference, a greater portion of individuals who received real TBS (1 mo: 80.8%; 2 mo: 77.0%; 3 mo: 73.1%) remained enrolled in the study, relative to sham (1 mo: 80.0%; 2 mo: 52.0%; 3 mo: 48.0%) at the 2-month (OR: 2.82, *z* = 1.692, *p* = .09) and 3-month (OR: 2.71, *z* = 1.659, *p* = .10) follow-up visits, which may be clinically meaningful (Figure 3). There was no significant difference in enrollment by gender.

Again, while there was not a statistically significant difference, a greater portion of individuals who received real TBS (1 mo: 53.3%; 2 mo: 60.0%; 3 mo: 73.3%) remained abstinent from alcohol relative to sham (1 mo: 52.9%; 2 mo: 47.1%; 3 mo: 47.1%) at the 3-month (OR: 3.09, *z* = 1.487, *p* = .14) follow-up visit, which may be clinically meaningful. There was no significant difference in sobriety by gender.

There was not a statically significant difference in return to heavy drinking in those who received real TBS (1 mo: 13.3%; 2 mo: 26.7%; 3 mo: 13.3%), relative to sham (1 mo: 17.6%; 2 mo: 23.5%; 3 mo: 17.6%). Further, there was no significant difference in the time to first drink following completion of the TMS visits in the group receiving real TBS (23.3 ± 21.4 days) relative to sham (12.2 ± 20.1 days; *t*_14_ = 1.05, *p* = .321).

### OCDS and AUQ

**Figure 4:**
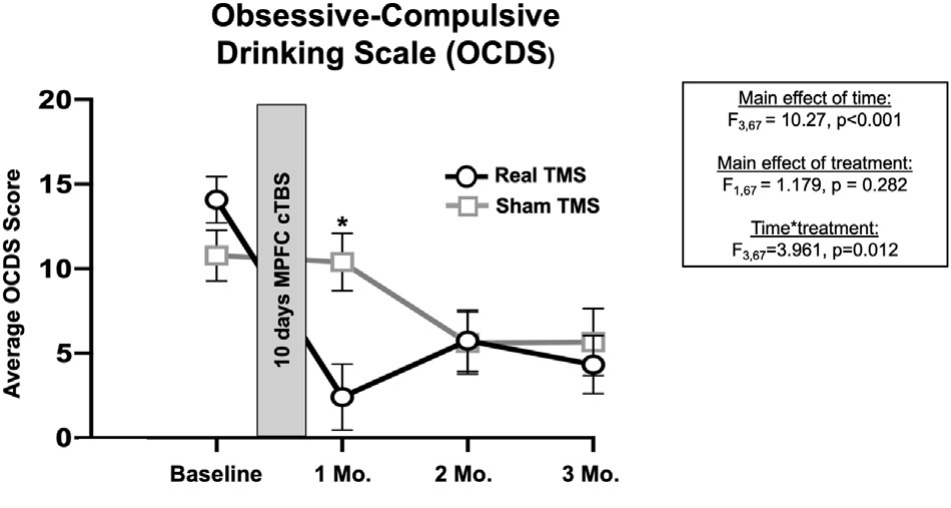
Alcohol craving (Obsessive-Compulsive Drinking Scale [OCDS]). There was a significant time × treatment interaction in obsessive-compulsive drinking scores that was driven by a significant reduction in OCDS scores in the real (black lines, circles) vs. sham group (gray lines, squares) 1 month after treatment (*t*_12_ = −3.065, *p* = .01, Hedges’ *g* = 1.65). Baseline OCDS score was a significant covariate in the model (*F*_1,67_ = 13.172, *p* < .001). Gender and scalp-to-cortex distance were not significant covariates. Results of general linear model analysis are embedded. Data plotted reflect estimated marginal means. Error bars represent SEM. cTBS, continuous theta burst stimulation; MPFC, medial prefrontal cortex; TMS, transcranial magnetic stimulation.

Analysis of the OCDS revealed a significant time × treatment interaction (*F*_3,67_ = 3.961, *p* = .012) and a main effect of time (*F*_3,67_ = 10.27, *p* < .001). Baseline OCDS score was a significant covariate in our model (*F*_1,67_ = 13.17, *p* < .001) (Figure 4). There was no significant effect of gender (*F*_1,67_ = 0.0872, *p* = .35) or STC distance (*F*_1,67_ = 0.267, *p* = .61). At the 1-month visit, estimated marginal means of OCDS score were significantly reduced in the real TBS group (2.4 ± 1.9) relative to sham (10.4 ± 1.7; *t*_12_ = −3.065; *p* = .01; Hedges’ *g* = 1.65). There was no main effect of treatment (*F*_1,73_ = 0.115, *p* = .74) or an interaction (*F*_3,73_ = 1.394, *p* = .25). Change in AUQ and OCDS subscales can be found in Figure S1.

### Change in Alcohol Cue–Induced FP1 Connectivity

**Figure 5:**
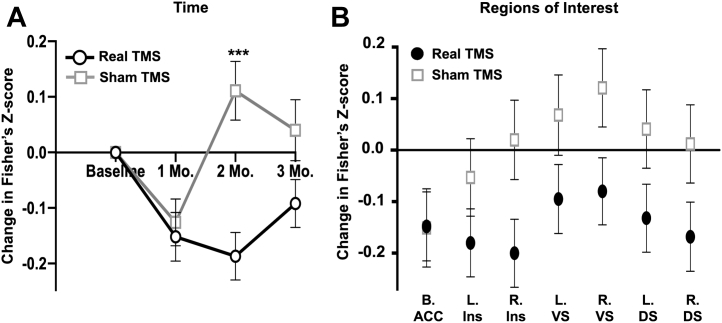
Influence of real vs. sham theta burst stimulation (TBS) over time and by region of interest. Estimated marginal means across time **(A)** and regions of interest **(B)** are plotted with respect to treatment (real TBS: black lines, circles; sham TBS: gray lines, squares). The general linear model assessing change in functional connectivity included covariates for scalp-to-cortex distance, gender, Beck Depression Inventory-II, Alcohol Use Disorders Identification Test, and trait anxiety. **(A)** There was a significant time × treatment interaction on frontal pole connectivity during alcohol cues (*F*_1,547_ = 4.519, *p* = .01). **(B)** Across all regions of interest, functional connectivity to alcohol cues was consistently lower in the group receiving real TBS relative to sham. Error bars represent SEM. ACC, anterior cingulate cortex; B, bilateral; DS, dorsal striatum; Ins, insula; L, left; R, right; VS, ventral striatum.

**Figure 6:**
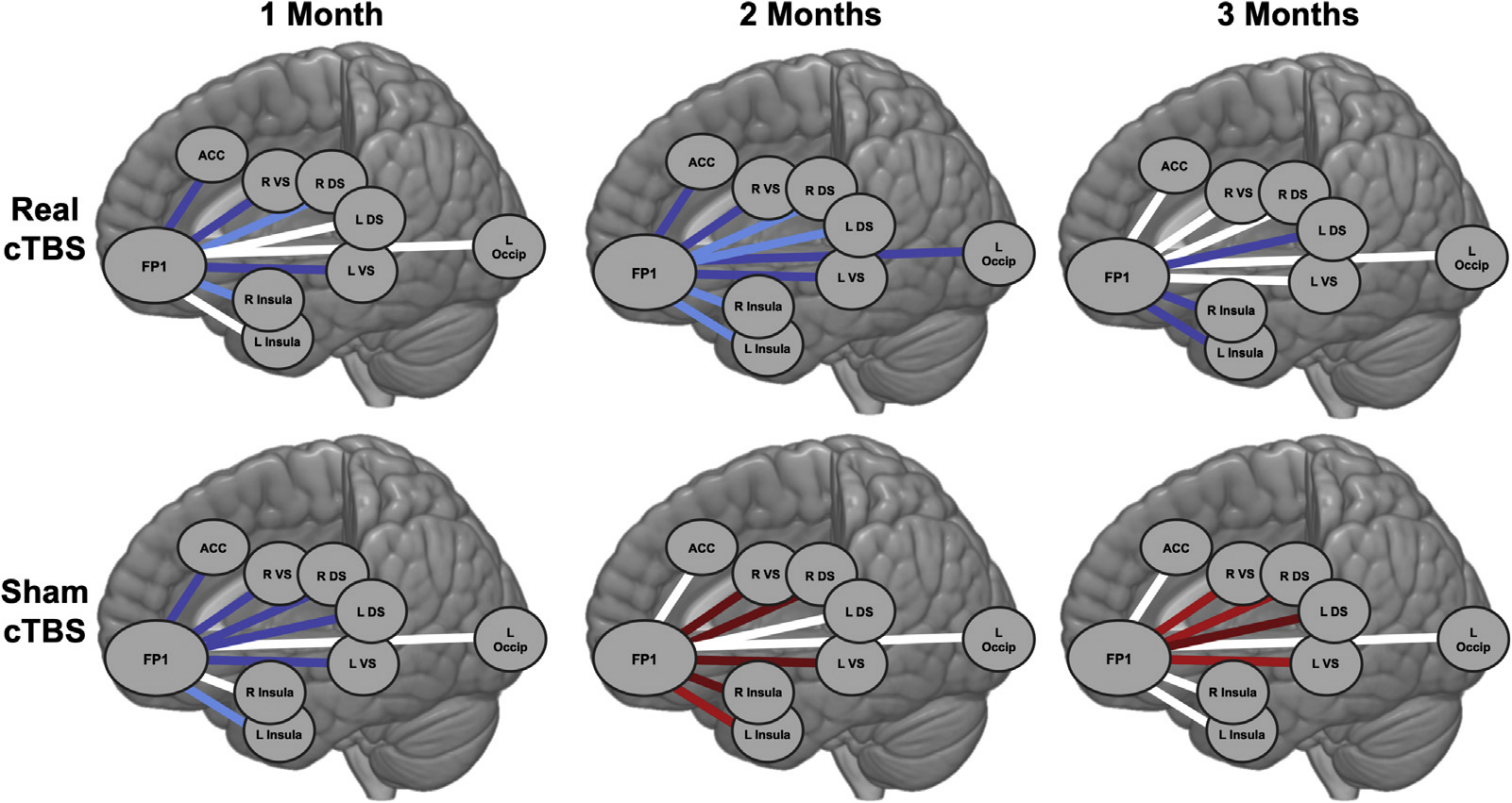
Change in cue-induced functional connectivity relative to baseline. The real theta burst stimulation (TBS) group had a reduction in connectivity 1, 2, and 3 months after the baseline visit. The sham TBS group had an initial reduction, but then became more reactive to alcohol cues at 2 and 3 months. Blue lines: lower connectivity relative to baseline; red lines: higher connectivity relative to baseline; white lines: no change in connectivity. Intensity of the lines reflect magnitude of functional connectivity change: dark blue/red indicates absolute change in correlation coefficient ≥ 0.1; bright blue/red indicates absolute change in correlation coefficient ≥ 0.2; white lines indicate absolute change in correlation coefficient < 0.1. ACC, anterior cingulate cortex; cTBS, continuous TBS; DS, dorsal striatum; FP1, frontal pole; L, left; Occip, superior occipital cortex; R, right; VS, ventral striatum.

There was a main effect of treatment (*F*_1,547_ = 14.235, *p* < .001), time (*F*_2,547_ = 3.823, *p* = .02), and time × treatment interaction (*F*_1,547_ = 4.519, *p* = .01). Gender emerged as a significant covariate (*F*_1,547_ = 5.279, *p* = .02). FP1 functional connectivity to the network of ROIs as a whole was significantly lower at the 2-month follow-up (*t*_25_ = −4.382, *p* < .001; Hedges’ *g* = 1.72), with the effects eroding at the 3-month follow-up (*t*_25_ = −1.914, *p* = .07; Hedges’ *g* = 0.75) (Figures 5A and 6). There was no treatment × ROI interaction (*F*_6,547_ = 0.540, *p* = .78), likely owing to the uniform effects of TBS on FP1 functional connectivity to these regions (Figure 5B).

### Secondary Analysis

**Figure 7:**
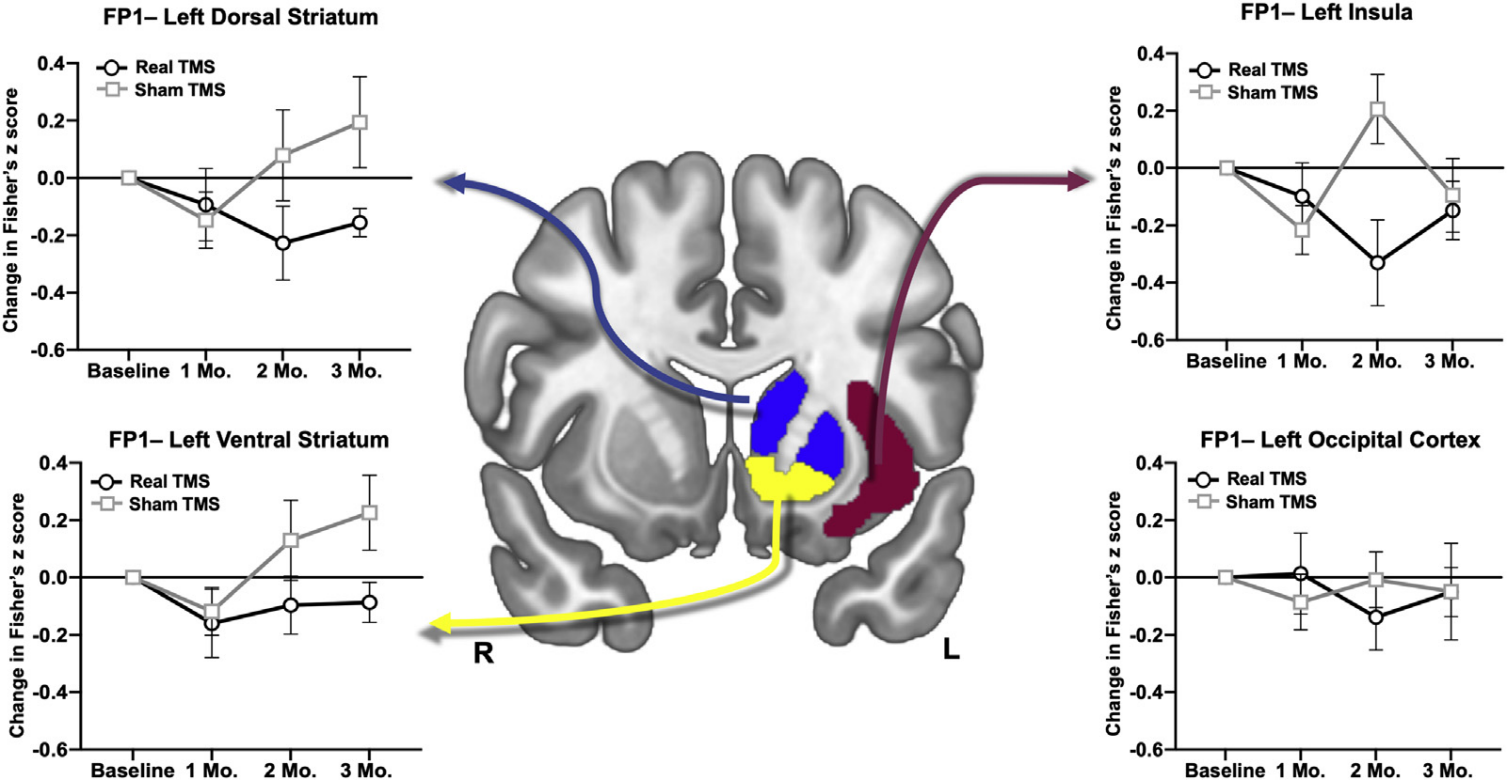
Longitudinal change in frontal-striatal and cingulate connectivity to alcohol cues. There was a substantial reduction in connectivity to alcohol cues following real (black lines, circles) vs. sham (gray lines, squares) theta burst stimulation to the left dorsal and ventral striatum as well as the left insula cingulate at 2 months. Dorsal striatal connectivity and ventral striatal connectivity to alcohol cues remained attenuated in the real theta burst stimulation group at 3 months. There was no difference in the occipital cortex, which served as a control region. Error bars represent SEM. See Table S2 for associated effect sizes. FP1, left frontal pole; TMS, transcranial magnetic stimulation.

Effect sizes were calculated for each ROI to inform future clinical trial design (Figure S2 and Table S2). Real TBS decreased functional connectivity from FP1 to the left dorsal striatum (2 mo: *g* = 0.589; 3 mo: *g* = 1.007), ventral striatum (2 mo: *g* = 0.527; 3 mo: *g* = 0.981), and left insula (2 mo: *g* = 1.046). There was no effect on connectivity to the left occipital cortex (Figure 7).

## Discussion

The last decade has brought about a groundswell of enthusiasm to advance TMS as a therapeutic option for individuals with substance use disorders. While the majority of clinical trials have focused on stimulating the left DLPFC, there is growing evidence that the FP1 may also be a fruitful treatment target given its transdiagnostic role in drug-cue reactivity (34). Our group recently demonstrated that a single session of TBS to the FP1 could decrease functional connectivity to drug and alcohol cues in a network of brain regions (e.g., dorsal and ventral striatum, cingulate, insula) (12). As a logical next step in the treatment development pipeline, we set out to determine if multiple sessions of TBS delivered to the FP1 could improve alcohol sobriety rates and brain reactivity to alcohol cues in a cohort of treatment-seeking individuals with AUD. The primary conclusions of this randomized, double-blind, sham-controlled longitudinal study are that 10 sessions of FP1 TBS 1) are well tolerated and a feasible addition to an intensive outpatient treatment program, 2) increased treatment engagement and sobriety 3 months after treatment initiation, and 3) produced a significant and durable decrease in alcohol cue–associated functional connectivity from the FP1 to the same network of regions that were modified in the single-session study (12). These data suggest that the FP1 continues to be a promising clinical treatment target for individuals with AUD and underscores the need for large multisite trials to evaluate this as a treatment adjuvant.

### Feasibility and Tolerability

Following the Food and Drug Administration clearance of TBS as a treatment tool for depression (35), there has been growing interest in developing high-potency forms of brain stimulation that can be delivered relatively quickly (36, 37, 38, 39). This high-density continuous protocol (12,40) not only was well tolerated over the FP1 but also resulted in a significant improvement in treatment engagement and alcohol sobriety in this sample. A recent study of 240 individuals that have come through our laboratory demonstrated that patient self-reported pain during TBS treatment over the FP1 was not statistically different than DLPFC stimulation (41). The integrity of our active sham condition was sound, with participant guesses regarding the received condition situated at near-chance levels.

### Current Use of Brain Stimulation in AUD

While the Food and Drug Administration and National Institute on Alcohol Abuse and Alcoholism have recently begun to expand the definition of a positive AUD treatment outcome to include a reduction in heavy drinking (42,43), achieving and maintaining abstinence remains a gold standard among treatment efforts for AUD. Further, it is well known that obsessive and compulsive drinking behaviors are key features of AUD (44). Here, we demonstrated that individuals who received real TBS were three times more likely to remain sober 3 months after treatment initiation relative to sham. This change in drinking behavior is preceded by a precipitous drop in obsessive and compulsive features of alcohol use 1 month after treatment, as well as a marked decrease in functional connectivity to alcohol cues 2 months after treatment. These data suggest that TMS may cause a cascade of changes in behavior and brain metrics, ultimately yielding an increase in sobriety 3 months after treatment.

These results are supported by early brain stimulation therapeutic work targeting the DLPFC, which reduced Alcohol Craving Questionnaire-Now scores (45,46) and alcohol consumption (47) following treatment. Our results most closely align with those of Ceccanti *et al.* (48) wherein, following 10 days of MPFC stimulation, alcohol consumption, craving, and brain reactivity to alcohol cues decreased. These data, in concert with previous literature, demonstrate that TBS applied to the MPFC is a durable and efficacious strategy to decrease alcohol use and alcohol-cue reactivity.

### Enrollment Across a Three-Month Study

Participant dropout rates from standard, psychosocial interventions for AUD are high (49). Further, dropout from AUD treatment is a robust predictor of future relapse to alcohol use (50), while adherence to treatment is associated with long-term improvement in AUD severity (51). While enrollment in treatment is a clinically relevant variable, many previous TMS-AUD trials have been unable to assess this metric because they occurred within an inpatient hospital setting, wherein attendance at treatment sessions was likely mandated (45, 46, 47,52,53). Here, we recruited individuals from an outpatient population, which offers the twofold benefit of 1) expanding the knowledge within the field regarding the influence of cTBS on longitudinal treatment engagement and 2) expanding potential treatment options for a larger portion of individuals with AUD outside the hospital setting.

In a recent TMS-AUD trial, wherein participants received 20-Hz deep MPFC repetitive TMS (without behavioral intervention), retention rates among those receiving real TMS at 2- and 3-month follow-up visits were between 40% and 50% (48). Here, in applying TBS as an adjuvant therapeutic to an intensive outpatient program, we demonstrate that, among those receiving real TBS, retention rates at the 2-month (77%) and 3-month (73%) follow-up visits were high compared with this previous TMS-only intervention. Among those receiving sham TBS, retention rates were in line with previous psychosocial and TMS-only efforts (52% and 48%, respectively). Taken together, these data suggest that TBS is a very effective tool in maintaining subject enrollment and therefore may be a fruitful strategy in improving longitudinal AUD treatment outcomes.

### Change in FP1 Alcohol Cue–Induced Functional Connectivity

Sophisticated preclinical tools, such as optogenetics, have been used to demonstrate that, in rodents, manipulation of the prelimbic cortex [functionally analogous to the human MPFC (54)] and downstream striatal targets changes alcohol and drug seeking behavior in a causal manner (55, 56, 57, 58). Converging clinical evidence has shown that brain reactivity to alcohol cues, specifically within frontostriatal circuitry, is a strong predictor of future relapse to alcohol (1, 2, 3, 4, 5, 6,8).

To this end, we sought to decrease frontostriatal connectivity in response to alcohol cues. Despite the translational promise of this goal, the existing literature using TMS to decrease brain response to alcohol cues within this circuit is sparse. In fact, few existing studies have pursued this strategy (12,52,59). Here, we replicate results from Kearney-Ramos *et al.* (12), wherein a single session of TBS reduced FP1 alcohol-induced functional connectivity to downstream targets such as the dorsal striatum, ventral striatum, and insula. We further demonstrate that 10 days of TBS produce a similar yet durable reduction in FP1 alcohol-induced functional connectivity to the same striatal and insular targets. These data lend further support for the use of TBS as a brain-based treatment for AUD.

While we observed a robust decrease in alcohol cue–induced functional connectivity, the magnitude of functional MRI blood oxygen level–dependent signal was not substantially reduced across the experiment (Figure S4). This likely reflects a change in the temporal dynamics of MPFC circuitry rather than a change in magnitude of alcohol cue–induced blood oxygen level–dependent signal. In line with these results, Herremans *et al.* (52) found no significant change in blood oxygen level–dependent signal magnitude during alcohol cues after 15 sessions of repetitive TMS (20 Hz, left DLPFC).

### Clinical Improvement in Obsessive-Compulsive Drinking

Another important observation is that this protocol decreased OCDS scores. This is a valuable addition to the growing literature pointing to the FP1 as a target for obsessive-compulsive disorder. Using a very similar protocol, 600 pulses of TBS directed to the FP1 (to target the orbitofrontal cortex), Price *et al.* (60) recently demonstrated that a single session of frontal pole TBS improved compulsive behaviors in a cohort of 69 individuals with obsessive-compulsive disorder and that these effects lasted for up to 1 week (60). Although the validity of the OCDS as a tool to predict future drinking behavior has been questioned (61), it is still a mainstay of alcohol treatment evaluation, likely due to many studies demonstrating its efficacy as a predictor of long-term outcome (15,62). Considered from the perspective of research domain criteria, this adds interest to the role of the FP1 and associated neural targets in mediating obsessive and compulsive behaviors more broadly.

### Limitations and Future Directions

While this study was not prospectively powered to assess the influence of gender, our statistical model revealed gender as a significant source of variance in functional connectivity change. Women receiving real TBS experienced the greatest initial reduction in alcohol cue–induced functional connectivity (Figure S5). This result is in line with recently published work form our group demonstrating that women have a shorter scalp-to-cortex distance at the FP1 and therefore receive a substantially stronger electrical field at this cortical target (29). Further, participant attrition and logistical issues at the MRI scanner reduced the number of usable scans to study functional connectivity, especially at follow-up visits. Because of this high dropout rate, we were unable to perform a robust statistical analysis of the relationship between change in behavior and alcohol cue–induced functional connectivity. To mitigate the influence of participant dropout in future clinical trials, the field might benefit from innovative strategies to improve participant retention in longitudinal alcohol treatment trials.

### Conclusions

To the best of our knowledge, this is the first randomized, double-blind, sham-controlled trial to deliver 10 days of TBS to a patient population. The primary preregistered outcome was the effect of 10 sessions of TBS on brain reactivity to cues (a logical extension of our prior single session TBS study). We observed a significant difference between the groups. A secondary outcome was the effect of this protocol on drinking. While we did not observe a statistically significant difference in sobriety, individuals that received real TMS were nearly three times as likely to remain sober. Future clinical trials properly powered to measure drinking as a primary end point are warranted.

Furthermore, this is the first randomized, double-blind, sham-controlled trial to deliver TBS to the FP1, a brain target that has garnered a lot of interest recently following the promising work from Price *et al.* (60) that demonstrated that TBS to this target could improve symptoms of obsessive-compulsive disorder. Finally, this TMS trial demonstrates that it is possible to improve alcohol treatment outcomes, drinking behavior, and brain reactivity to alcohol cues for up to 3 months after treatment initiation. Because unique forms of TMS are gaining Food and Drug Administration clearance for diseases such as obsessive-compulsive disorder and smoking cessation, this study is an important step forward in expanding the potential indications for treatment of AUD.

## Supplementary Material

Supplementary Material

Key Resources Table
