# Supplementary material for: Medial Prefrontal Cortex Theta Burst Stimulation Improves Treatment Outcomes in Alcohol Use Disorder: A Double-Blind, Sham-Controlled Neuroimaging Study
Source: Biol Psychiatry Glob Open Sci. 2022 Mar 15;3(2):301–10. doi: 10.1016/j.bpsgos.2022.03.002 (PMC10140456; doi:10.1016/j.bpsgos.2022.03.002)
Supplement: Supplementary Material [file mmc1.pdf]

Medial Prefrontal Cortex Theta Burst Stimulation Improves Treatment Outcomes in Alcohol Use Disorder: A Double-Blind, Sham-Controlled Neuroimaging Study

Supplemental Information

Extended participant exclusion criteria: Exclusion criteria included current or prior dependence on prescription or psychoactive drugs other than nicotine (current smokers n=26), history of head injury with loss of consciousness, unstable medical illness, pregnancy or breast feeding, ferromagnetic metal in the body, history of seizures, and a Clinical Institute Withdrawal Assessment score > 5. Prior to each study visit, participants received a multi-panel urine drug screen (QuikVue 6-panel urine drug screen; Quidel, San Diego, CA). TMS was not delivered to individuals if they had a positive UDS for amphetamine (1000 ng/mL), cocaine (300 ng/mL) barbiturates (300 ng/mL), opiates (morphine 300ng/mL), tetrahydrocannabidiol (50 ng/mL), or benzodiazepines (300 ng/mL). If an individual was positive form one of these substances, they were allowed to reschedule the visit.

Intensive Outpatient Programming: The Intensive Outpatient Program consists of 20 total treatment days (3 hours/day). Participants attend daily group therapy sessions with clinicians trained in several different modalities of evidence-based treatment for alcohol use disorders (e.g., Motivational Enhancement Therapy, Cognitive Behavioral Therapy, Twelve Step Facilitation, and Acceptance and Commitment Therapy). There is a Day program (Mon-Fri, 9am-12pm) and an Evening Program (Mon-Thu, 530pm-830pm). Of the 51 individuals in this study 24 were enrolled in the Day program and 27 were enrolled in the Evening program. 27 of the 51 participants received their daily TBS sessions before their IOP sessions (typically within 1 hour). 24 participants received TBS after their IOP visits (all of whom were in the Day program). From a conceptual perspective it is possible that delivery of the TBS before versus after the IOP therapy sessions may influence treatment outcomes. Although there are not enough data points to evaluate this hypothesis in the present experiment, there did not appear to be a qualitative difference in returning to drinking rates between the individuals in the Day program that received real TBS before their IOP visits (66%) versus those that received TBS after their IOP visits (50%) at the 3-month time point.

Neuroimaging sample size: Of the 51 randomized participants, neuroimaging data was available from 44 individuals (real cTBS n=23; sham cTBS n=21). 7 individuals were not scanned due to a scanner upgrade. Within the real cTBS group, there were 23, 17, 16, and 16 fMRI scans available and within the sham group there were 21, 17, 11, and 11 scans available at baseline, 1, 2 and 3 month visits, respectively.

fMRI Preprocessing: All MRI data were processed using SPM12 (Wellcome Department of Cognitive Neurology, London, UK) implemented in MATLAB 2017b (MathWorks, Inc, Natick, MA). Before processing, images were converted from dicom to 4D NifTii format. Structural, T1-weighted images were segmented into their component parts (Gray matter, white matter, CSF, skull, and background), bias corrected, and normalized simultaneously (*SPM: segment*). Gray matter, white matter and CSF tissue segmentations were then applied as a combined mask to remove the skull from the anatomical image (*SPM: imcalc)*. Field map derived voxel displacement maps were generated and co-registered to the functional images (*SPM: VDM*). Functional images were then realigned with rigid-body 6 motion parameter realignment to the first image in the series using a least-squares approach. Previously

calculated voxel displacement maps were used to unwarp the functional images, reducing image distortion and preserving anatomical fidelity (*SPM: Realign and Unwarp)*. The functional images were then co-registered, through the mean T2* image, to the skull-stripped anatomical (*SPM: Coregister: Estimate*). The co-registered images were then normalized to standard MNI-152 space using the non-linear deformation maps obtained during segmentation. Lastly, functional images were skull-stripped and smoothed with an 8mm FWHM kernel to enhance the signal-to-noise ratio and reduce inter-subject variability.

Alcohol cue-reactivity (General Linear Modeling, whole-brain approach): All whole-brain alcohol cue-reactivity analyses were performed in SPM12. Briefly, for each participant, smoothed, normalized functional data from each condition (alcohol, neutral beverages, blurred and fixation cross cues) were concatenated. Motion parameters in 6 directions (x,y,z, pitch, roll, and yaw) were treated as nuisance variables and regressed out of the data. Motion-adjusted contrast maps representing relative beta weights were created for the alcohol cue-condition (as well as the neutral beverage, blur, and fixation cross conditions). To assess fMRI BOLD response to alcohol cues on the group level, a general linear model (GLM) of the alcohol cue contrast maps was performed. A full-factorial model (adjusting for subject dropout) was constructed with group (real or sham TBS) and time (Baseline, 1 Month, 2 Months, and 3 months follow-ups) as fixed factors. Real and sham samples were assumed to be independent, however within group follow-up scans were considered dependent. Variance was assumed to equal between the groups at each timepoint. A binary, explicit MNI mask (encompassing gray matter, white matter and CSF) was applied to the data. Individuals with greater than 3mm of motion in any plane were excluded from this analysis. Contrasts comparing group-level beta weights within as well as between groups (e.g. real only, sham only, and sham>real) were generated at each visit. Second level results were assessed using a voxel-wise threshold of p=0.05 and cluster-level threshold (k) was corrected for each second level map such that only clusters surviving Family-Wise Error multiple comparisons corrected remained.

Scalp-to-cortex distance: SIMNIBS v3.2.1 measures the shortest distance between the cortex and the area immediately underlying the TMS coil. This measurement can be found within SIMNIBS' standard output file titled "simnibs_simulation_date_time.log". Data are initially reported as coil-cortex distances. For this manuscript, the distance modeled from the coil to the skin (4mm) was subtracted from each value to produce scalp-to-cortex distances.

Change in depression (BDI), anxiety (STAI), and impulsivity (BIS): Mixed effects general linear models (time*treatment) were constructed for each variable: BDI, STAI state, STAI trait, and BIS.

Fig S1: Alcohol craving (AUQ and OCDS sub-scales)

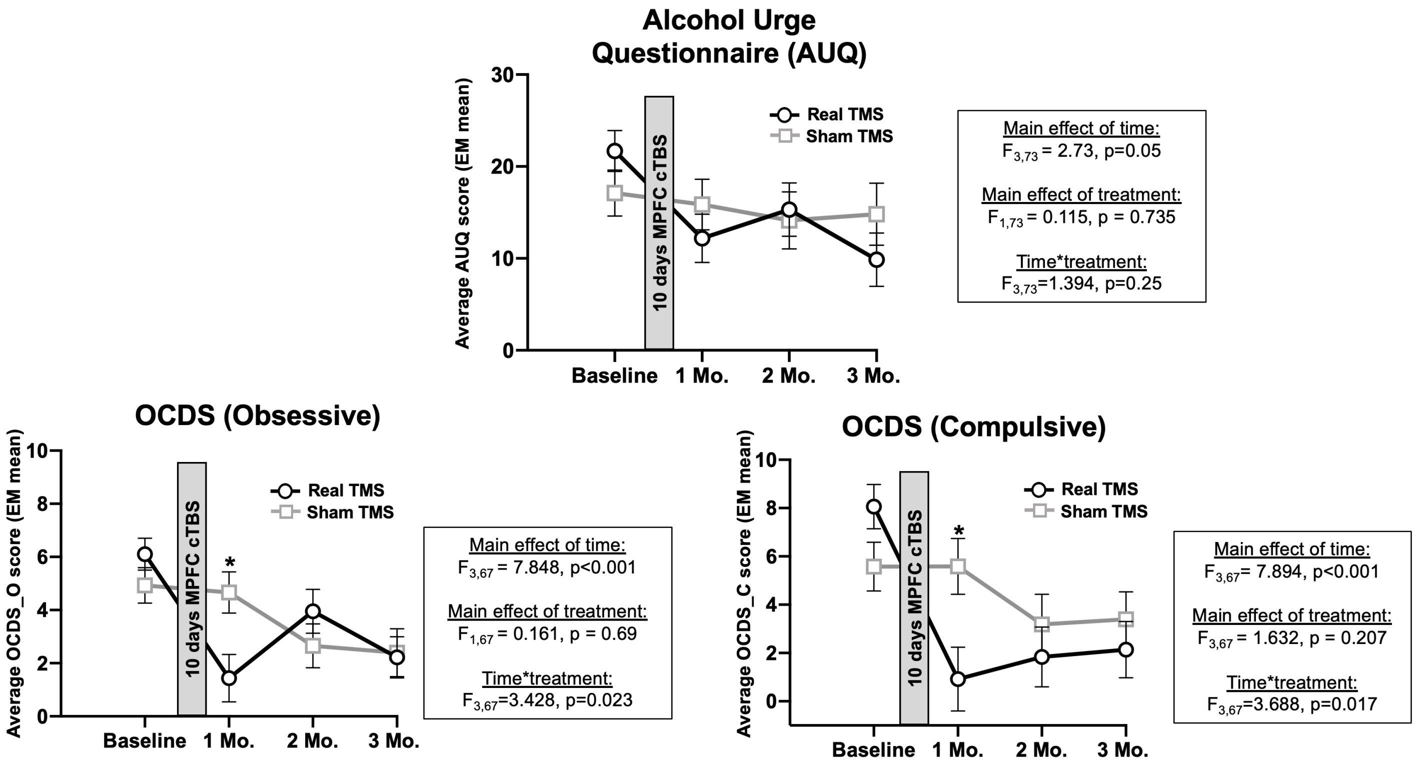

Figure S1: Alcohol craving (AUQ and OCDS sub-scales). Average AUQ, OCDS obsessive, and OCDS compulsive subscales were measured at each study visit. Black lines (circles) represent those receiving cTBS; grey lines (squares) represent those receiving sham. Data plotted are estimated marginal means, with covariates for gender, scalp-to-cortex distance, and baseline score appearing in the model. AUQ score decreased in both groups across the experiment but was not significantly different at any visit. Baseline AUQ score was a significant covariate ($F_{1,73}$=24.80, p<0.001). OCDS obsessive and compulsive subscales were significantly reduced immediately following real cTBS, relative to sham cTBS (1 month, Obsessive: Real cTBS, 1.40 ± 0.90; Sham cTBS, 4.66 ± 2.19) t_{12}=-2.76, p=0.02, hedge's g=1.49; 1 month, Compulsive: Real cTBS, 0.92 ± 1.32; Sham cTBS, 5.59 ± 3.26) t_{12}=-2.66, p=0.02, hedge's g=1.41). Baseline score was a significant covariate in both models (OCDS_O: $F_{1,67}$=18,43, p<0.001; OCDS_C: $F_{1,67}$=11.34, p=0.001). Gender and scalp-to-cortex distance were not significant covariates. Results of general linear model analysis are embedded. Error bars represent SEM.

Figure S2: Magnitude of alcohol cue-induced functional connectivity change (all ROIs)

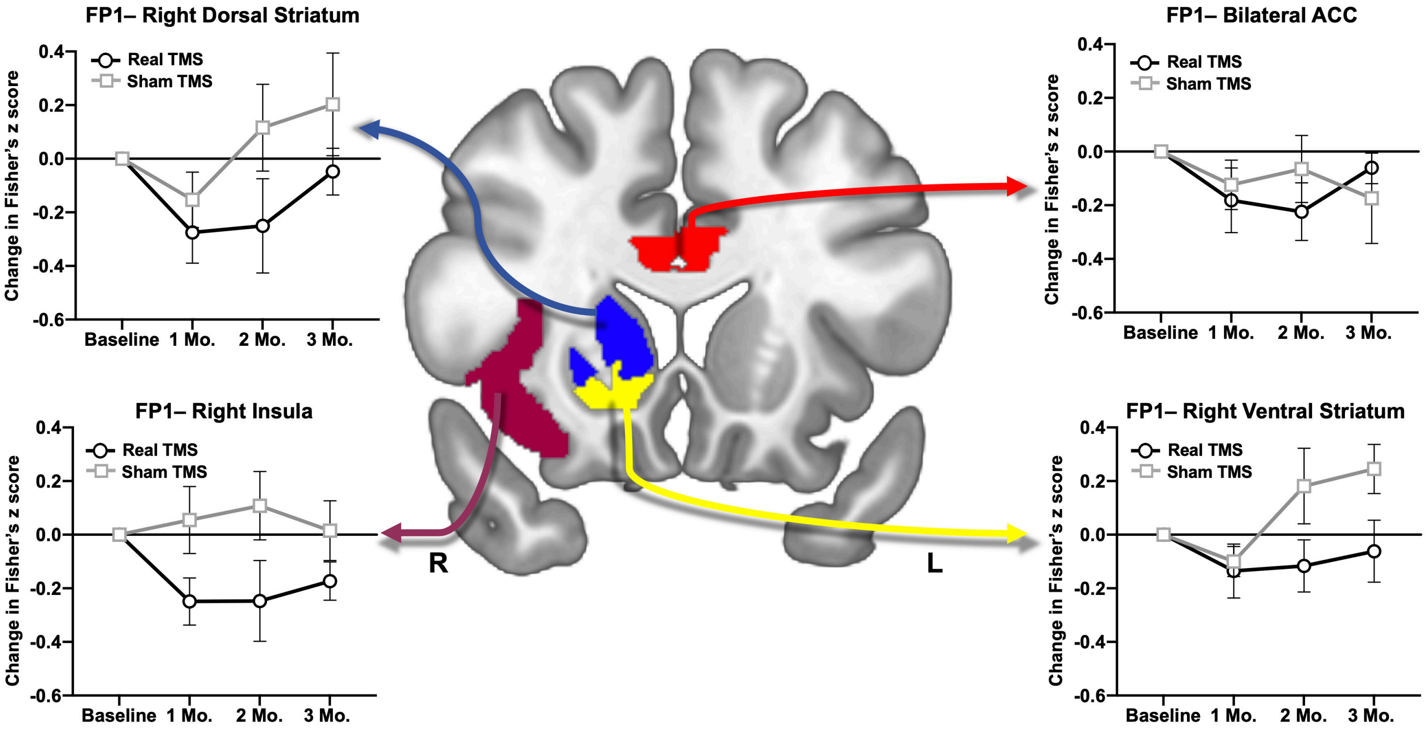

Figure S2: Magnitude of alcohol cue-induced functional connectivity change (all ROIs). There was an overall reduction in FP1 connectivity following real TBS (black lines, circles) relative to sham TBS (grey lines, squares). Data plotted represent change in Fisher's transformed correlation coefficient (z-scores). Error bars represent SEM. See Table S2 for relevant effect sizes.

Figure S3: Behavioral variables across experiment.

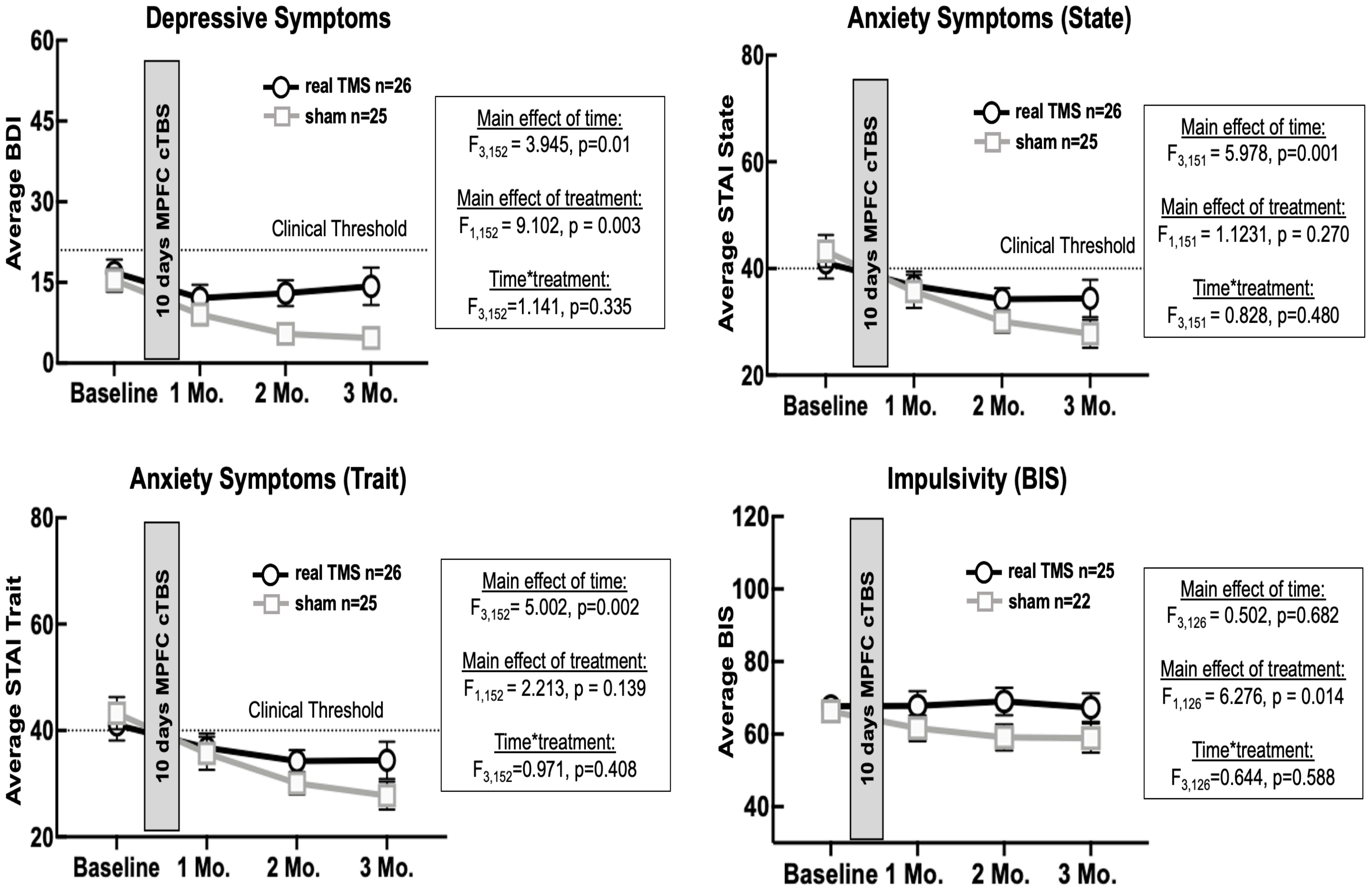

Figure S3: Behavioral variables across experiment. Average BDI, STAI (state and trait sub-scores) and BIS were measured at each study visit. Black lines (circles) represent real cTBS; grey lines (squares) represent sham cTBS. Dotted lines on Y axis represent clinical thresholds for severe depression and anxiety. Average values for depression and anxiety severity remained at or below clinical thresholds throughout the duration of the experiment. Results of general linear model analysis are embedded. Error bars represent SEM.

Figure S4: Alcohol Cue Reactivity (General Linear Model Analysis)

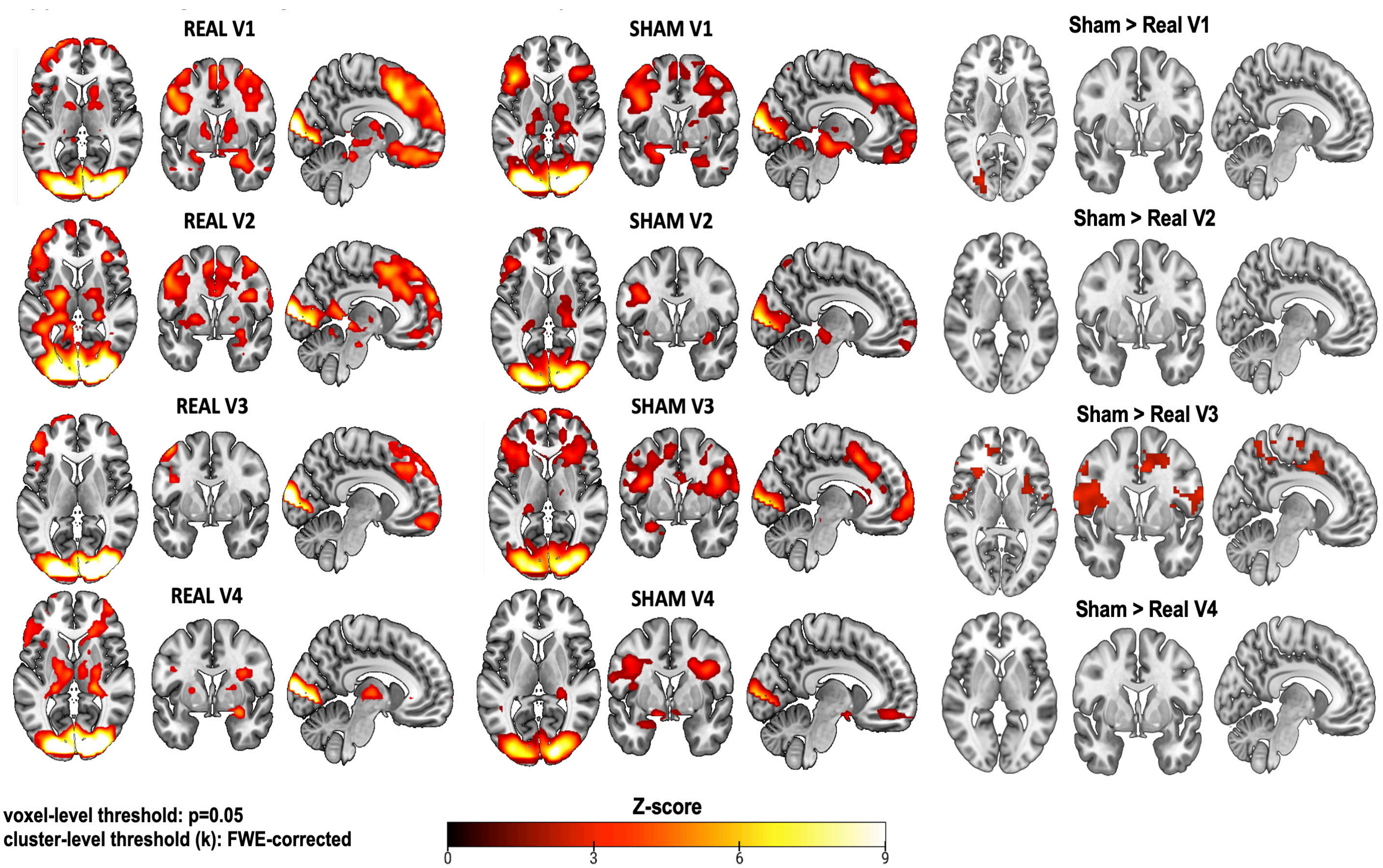

Figure S4: Alcohol cue reactivity (General Linear Model analysis). There was no significant difference in alcohol cue-reactivity between the Real and Sham groups at the baseline (V1), 1 month (V2) or 3-month (V4) visits ($p<0.05$, family-wise error multiple comparisons-corrected). At the 2-month visit (V3), individuals in the Sham group had greater alcohol cue-reactivity than individuals in the Real TMS group. The difference (sham > real) in cue-reactivity was greatest in two clusters (cluster 1: $k=2549$, $p=0.004$; cluster 2: $k=2529$, $p=0.004$). These differences were detected within the right insula and right precentral gyrus (cluster 1), and the left mid cingulum and left postcentral gyrus (cluster 2).

Figure S5: Influence of Gender on change in alcohol cue-reactivity.

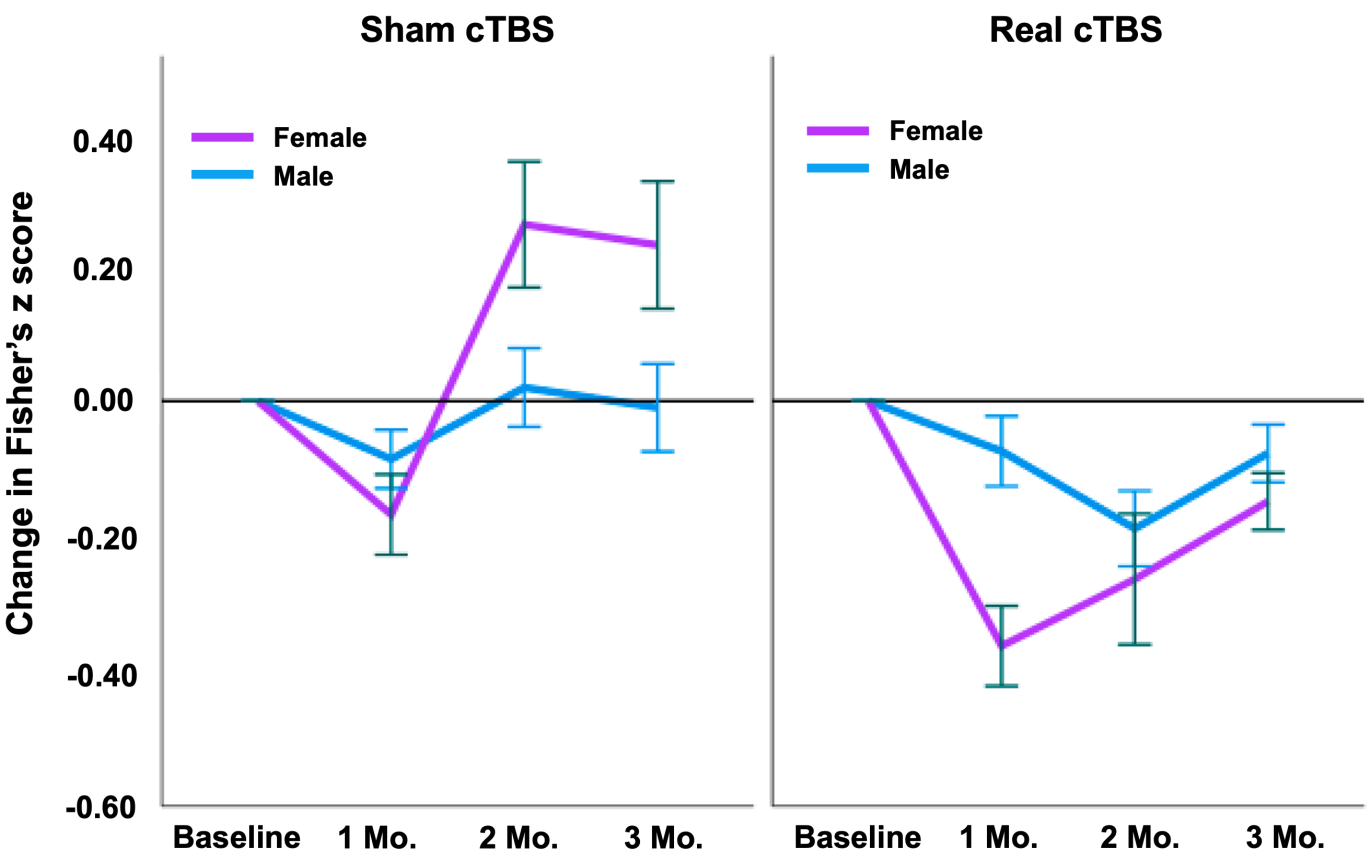

Figure S5: Influence of Gender on change in alcohol cue-reactivity. Women (purple lines) receiving sham cTBS (left panel) experienced a greater increase in alcohol cue-induced functional connectivity at the 2- and 3-month follow-up visits, relative to men (blue lines). Conversely, women receiving real cTBS (right panel) experienced a greater reduction in alcohol cue-induced functional connectivity relative to men (blue lines). Error bars reflect SEM.

Table S1: Outlier quantification in alcohol cue induced FP1 functional connectivity.

		1 Month	2 Months	3 Months
**Real cTBS**	**Total # of data points**	**136**	**128**	**128**
	**Total Outliers**	**6 (0, 6)**	**4 (2, 2)**	**3 (0, 3)**
	**% of data points analyzed**	**96%**	**97%**	**98%**
**Sham cTBS**	**Total # of data points**	**136**	**88**	**88**
	**Total Outliers**	**2 (1, 1)**	**0 (0, 0)**	**3 (2, 1)**
	**% of total data points analyzed**	**99%**	**100%**	**97%**

Table S1: Outlier quantification in alcohol cue-induced FP1 functional connectivity. Outlier data points among change in FP1 connectivity were removed from analysis as described. Number of outlier data points are listed as Total (# low outliers, # high outliers). Of 704 collected datapoints, 18 (2.5%) were deemed as extreme outliers and removed from analysis.

Table S2: Change in FP1 functional connectivity to all tested ROIs (effect sizes).

Seed	Source	Effect size at 1 Mo.	Effect size at 2 Mo.	Effect Size at 3 Mo.
Left Frontal Pole (FP1)	L Dorsal Striatum	0.119	0.589	1.007
	R Dorsal Striatum	0.281	0.586	0.543
	L Ventral Striatum	0.107	0.527	0.981
	R Ventral Striatum	0.109	0.705	0.755
	L Insula	0.294	1.046	0.127
	R Insula	0.707	0.847	0.595
	B. Anterior Cingulate	0.137	0.383	0.301
	Left Occipital Cortex	0.204	0.339	0.004

Table S2: Change in FP1 functional connectivity to all tested ROIs (effect sizes). Effect sizes (Hedge's g) reflecting the difference in functional connectivity change in real, relative to sham TBS, are shown for each ROI, at each timepoint. Length of the overlaid blue bars reflect the magnitude of effect size in difference between real and sham group (scaled from 0.0 to 1.046).
